# Protocol to study phagocytosis of apoptotic murine splenocytes by syngeneic antigen-presenting cells *in vitro*

**DOI:** 10.1016/j.xpro.2025.103903

**Published:** 2025-06-14

**Authors:** Lukas M. Braun, Robert Zeiser

**Affiliations:** 1 Department of Internal Medicine I, Medical Center – University of Freiburg, Faculty of Medicine, University of Freiburg, Freiburg, Germany; 2 German Cancer Consortium (DKTK), Partner Site Freiburg, a Partnership Between DKFZ and Medical Center – University of Freiburg, Freiburg im Breisgau, Germany; 3 Signalling Research Centres BIOSS and CIBSS – Centre for Integrative Biological Signalling Studies, University of Freiburg, Freiburg, Germany

**Keywords:** Cancer, Immunology, Microscopy

## Abstract

Extracorporeal photopheresis (ECP) effectively reduces immune checkpoint inhibitor (ICI)-induced colitis and is based on the phagocytic uptake of apoptotic ECP-treated splenocytes. Here, we present a flow cytometry-based assay to investigate phagocytosis of ECP-treated murine splenocytes by bone marrow-derived macrophages and dendritic cells *in vitro* using a congenic marker only present in donor animals and fluorescence labeling of ECP-treated cells. We describe steps for analyzing M2-like polarization markers, investigating protein phosphorylation, and analyzing specific target gene expression in single cells.

For complete details on the use and execution of this protocol, please refer to Braun et al.^1^

## Highlights

• FACS-based phagocytosis analysis of fluorescently labeled cells by APCs *in vitro*
• Analysis of BMDM polarization after co-culture with apoptotic cells
• FACS-based investigation of target gene expression on single-cell level
• Steps to induce cell death with extracorporeal photopheresis

## Abstract (editor highlights)

Publisher’s note: Undertaking any experimental protocol requires adherence to local institutional guidelines for laboratory safety and ethics.

## Graphical abstract

**Figure:**
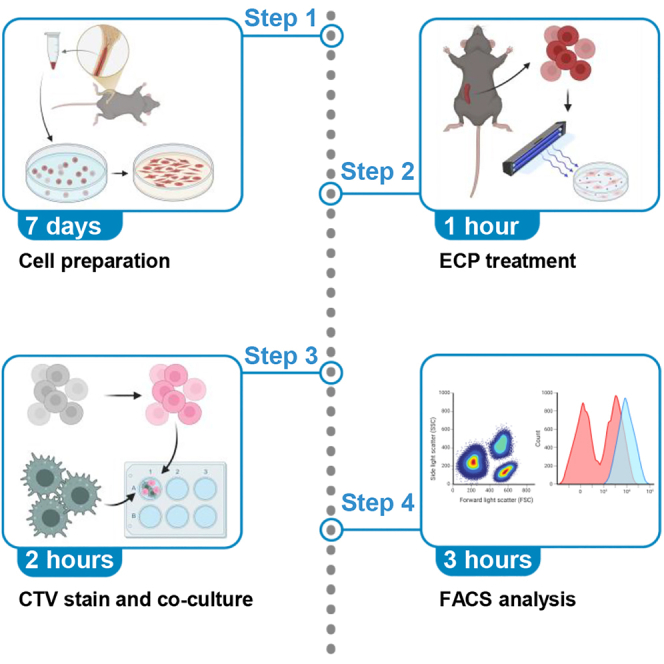

## Before you begin

The protocol presented below was developed to investigate the phagocytic uptake of Extracorporeal Photopheresis (ECP)-treated splenocytes by bone marrow-derived macrophages or bone marrow-derived dendritic cells. The development of this protocol was based on our finding that ECP-treated splenocytes are a powerful therapeutic tool to treat inflammatory side effects of immune checkpoint inhibition, mainly colitis, in cancer patients. ECP describes the treatment of immune cells with 8-methoxypsoralen and UVA, thereby inducing apoptosis in these cells. In our preclinical model, we have established splenocytes as immune cell pool for ECP therapy and ECP was used as a therapy to treat immune checkpoint inhibitor (ICI)-induced inflammatory side effects.^1^ Besides inducing cell death, we wanted to understand if phagocytes clear these ECP-treated apoptotic splenocytes. In addition, we aimed to understand if the phagocytic uptake of ECP-treated splenocytes results in immunosuppressive polarization and Adiponectin expression.

This protocol describes the steps to investigate the phagocytic uptake of syngeneic splenocytes by antigen presenting cells (APCs) using flow cytometry. The splenocytes were treated with ECP to induce apoptosis. To understand if ECP-treated splenocytes undergo apoptosis and if these apoptotic cells are engulfed by APCs, we have co-cultured ECP-treated splenocytes with bone marrow-derived macrophages (BMDMs) or bone marrow-derived dendritic cells (BMDCs). We use male and female C57BL/6N (CD45.2, Janvier Labs) and CD45.1 (Charles River) mice with an age of 7–10 weeks in the following protocol. The use of CD45.2 mice for the culture of BMDMs and BMDCs and the use of CD45.1 mice for splenocyte isolation enables us to discriminate between ECP-treated cells and APCs. In addition, staining of ECP-treated splenocytes with a fluorescent dye (CellTrace Violet, CTV) helps us to understand if these splenocytes are phagocytosed by APCs upon co-culture for 48 h. Upon staining of surface markers and intracellular proteins, we could understand if the phagocytic uptake of ECP-treated splenocytes renders the APCs towards a more inflammatory or more tolerogenic phenotype. The staining of CD45.1 marks ECP-treated splenocytes, whereas CD45.2 stains BMDMs and BMDCs (phagocytes). Before surface antibody staining, the Fc receptors are blocked using a purified anti-CD16/32 antibody to prevent unspecific antibody binding. In the presented protocol, we have focused the analysis of APC differentiation mainly on BMDMs, but any surface markers for BMDCs can be included into the analysis. Moreover, the PhosFlow analysis performed in this protocol only included phosphorylation of STAT6. Alternatively, staining for any other phosphorylated proteins can be incorporated if needed for analysis. The same accounts for intracellular proteins, which only included analysis of Arginase 1 in this protocol. Phosphorylation of STAT6 and expression for Arginase 1 indicate, together with surface expression of CD206 and CD301, the polarization of macrophages towards and immunosuppressive M2-like phenotype. Further, we describe a method to investigate gene expression in single cells by flow cytometry, using the PrimeFlow method. This method can be extended to analyze more transcripts; however, a limited number of fluorophores provided by the manufacturer restricts the panel design. In addition, we present a protocol for the staining of surface calreticulin, which acts as an engulfment signal for phagocytic cells.^2^^,^^3^

### Institutional permissions

Any experiments on live vertebrates or higher invertebrates must be performed in accordance with relevant institutional and national guidelines and regulations. Animal studies were carried out in compliance with relevant animal-use guidelines and ethical regulations. The protocol was approved by the Regierungspräsidium Freiburg, Germany (Protocol approval number: X-20/06K).

C57BL/6 mice (CD45.2) were purchased either from Janvier Labs (France) or obtained from the local stock of the animal facility at University Medical Center Freiburg (Germany). B6.SJL-*Ptprc*^*a*^*Pepc*^*b*^/BoyCrl (Ly5.1, CD45.1) mice were originally purchased from Charles River. All mouse strains were bred at the local animal facility at University Medical Center Freiburg (Germany). All mice were housed under specific pathogen-free conditions at the University Medical Center Freiburg. Mice were used between 7 and 12 weeks of age, and only female or male donor/recipient pairs were used.

### Preparation of culture medium and buffers

**Timing: 1 h**

1. Prepare cRPMI, staining buffer and buffers for PrimeFlow before use.
2. cRPMI and flow cytometry staining buffer can be stored at 4°C. Buffers for PrimeFlow should be prepared directly before use.
3. Recombinant murine M-CSF and recombinant murine GM-CSF are bought as lyophilized stock and should be prepared according to manufacturer’s instructions. The LOT-specific instructions for cytokine reconstitutions can be downloaded after the purchase of the product.
  a. Cytokine stocks can be stored at −20°C.
  b. Cytokines should always be added freshly to the culture medium.

### Bone marrow isolation

**Timing: 1 h**

The harvest of bones and the bone marrow isolation should be performed according to established procedures, which can differ between labs. If researchers have limited experience with harvesting bones and isolating bone marrow, the procedure is described in details with pictures in previously published protocols.^4^^,^^5^

4. Euthanize the donor mice (C57BL/6, CD45.2) according to institutional guidelines.
5. Disinfect the mice with 70% ethanol.
6. Isolate femur and tibia, remove muscles and attached tissue and place the bones in cold 1X PBS; the hips can be isolated if higher cell numbers are needed.^5^
7. Under a sterile hood, flush the bone marrow into 1X PBS using a syringe and needle or any other method of choice.

**CRITICAL:** From here on, all steps should be performed under a laminar flow hood with sterile equipment.

8. Resuspend cells and filter through a 70 μm strainer into a 50 mL tube.
9. Spin cells down at 300 × *g* for 10 min at 4°C.
10. Discard supernatant and resuspend the pellet in 2 mL erythrocyte lysis buffer (e.g. ACK lysis buffer).
  a. Incubate for 2 min at 20–25°C (RT).
  b. Add 40 mL 1X PBS.

**CRITICAL:** Erythrocyte lysis is important, as remaining erythrocytes are toxic to stem cells in the culture.

11. Spin cells down at 300 × *g* for 10 min at 4°C.
12. Discard the supernatant.
13. Resuspend cells in 30 mL cRPMI and count the cells.

## Key resources table

**Table:** 

REAGENT or RESOURCE	SOURCE	IDENTIFIER
**Antibodies**		

Anti-mouse/human Arginase 1 (APC; clone A1exF5; dilution 1:100)	Thermo Fisher Scientific	Cat# 17-3697-82; RRID:AB_2734835
Anti-mouse CD16/32 (clone 93; dilution 1:25)	BioLegend	Cat# 101302; RRID:AB_312801
Anti-mouse CD45.1 (FITC; clone A20; dilution 1:100)	BioLegend	Cat# 110706; RRID:AB_313494
Anti-mouse CD45.2 (BV711; clone 104; dilution 1:100)	BioLegend	Cat# 109847; RRID:AB_2616859
Anti-mouse CD45.2 (PE; clone 104; dilution 1:100)	BioLegend	Cat# 109808; RRID:AB_313444
Anti-mouse CD206 (PE; clone C068C2; dilution 1:100)	BioLegend	Cat# 141706; RRID:AB_10895754
Anti-mouse CD301 (PE/Cy7; clone LOM-14; dilution 1:100)	BioLegend	Cat# 145705; RRID:AB_2562939
Anti-mouse Stat6, pY641 (Alexa Fluor 488; clone J71-773.58.11; dilution 1:5)	BD Biosciences	Cat# 558243; RRID:AB_647099
Calreticulin (dilution 1:200)	Cell Signaling Technology	Cat# 2891; RRID:AB_2275208
F(ab')2-donkey anti-rabbit IgG (H+L) secondary antibody (PE; dilution 1:50)	Thermo Fisher Scientific	Cat# 12-4739-81; RRID:AB_1210761
OVA257-264 (SIINFEKL) peptide bound to H-2Kb (PE; clone 25-D1.16; dilution 1:100)	Thermo Fisher Scientific	Cat# 12-5743-82; RRID:AB_925774

**Chemicals, peptides, and recombinant proteins**		

8-methoxypsoralen (UVADEX)	Therakos	PZN# 01087204
ACK lysing buffer	Thermo Fisher Scientific	Cat# A1049201
BD Phosflow Lyse/Fix buffer 5X	BD Biosciences	Cat# 558049
BD Phosflow Perm buffer III	BD Biosciences	Cat# 558050
CellTrace Violet	Thermo Fisher Scientific	Cat# C34557
EDTA (0.5 M), pH 8.0	Thermo Fisher Scientific	Cat# AM9261
Fetal bovine serum (Fetal Constance, EU approved)	Anprotec	Cat# AC-SM-0190
Murine GM-CSF (recombinant)	Thermo Fisher Scientific	Cat# 315-03-100UG
Murine M-CSF (recombinant)	Thermo Fisher Scientific	Cat# 315-02-100UG
Ovalbumin (257–264) chicken	Sigma	Cat# S7951-1MG
Penicillin-streptomycin	Thermo Fisher Scientific	Cat# 15140122
RPMI 1640	Thermo Fisher Scientific	Cat# 21875034
Zombie NIR fixable viability kit	BioLegend	Cat# 423106

**Critical commercial assays**		

Adipoq-specific Alexa Fluor 647 labeled probe, type 1	Thermo Fisher Scientific	Cat# VB1-17726-PF
Cytofix/Cytoperm fixation/permeabilization kit	BD Biosciences	Cat# 554714
PrimeFlow RNA assay kit	Thermo Fisher Scientific	Cat# 88-18005-210

**Experimental models: Organisms/strains**		

Mouse: C57BL/6N (CD45.2) 7–12 weeks, male and female	Janvier Labs	RRID:IMSR_RJ:C57BL-6NRJ
Mouse: CD45.1: B6.SJL-*Ptprc*^*a*^*Pepc*^*b*^/BoyCrl 7–12 weeks, male and female	Charles River	Strain #494; RRID:IMSR_CRL:494

**Software and algorithms**		

FlowJo v.10	BD Biosciences	N/A
FACSDiva software v.6	BD Biosciences	N/A
GraphPad Prism v.10	GraphPad	N/A

**Other**		

BS-02 UV irradiation chamber equipped with UVA	Opsytec Dr. Gröbel	Cat# 860902; 860820

## Materials and equipment

**Table:** Basic medium for BMDM and BMDC culture (cRPMI)

Reagent	Final concentration	Amount
RPMI	N/A	445 mL
FCS (heat inactivated)	10%	50 mL
Penicillin/ streptomycin (10,000 units/mL)	100 units/mL	5 mL
**Total**	N/A	**500 mL**

Store at 4°C for a maximum of 2 months. Pre-warm medium to 37°C before use.

**Table:** BMDM differentiation medium

Reagent	Final concentration	Amount
cRPMI	N/A	10 mL
Murine M-CSF (20 μg/mL)	20 ng/mL	10 μL
**Total**	N/A	**10 mL**

Prepare directly before use. M-CSF should be added into the pre-warmed medium (37°C).

**Table:** BMDC differentiation medium

Reagent	Final concentration	Amount
cRPMI	N/A	10 mL
Murine GM-CSF (40 μg/mL)	40 ng/mL	10 μL
**Total**	N/A	**10 mL**

Prepare directly before use. GM-CSF should be added into the pre-warmed medium (37°C).

***Alternatives:*** GM-CSF can be used as recombinant protein or supernatant from hybridoma supernatant.

**Table:** Flow cytometry staining buffer

Reagent	Final concentration	Amount
dH_2_O	N/A	435.5 mL
PBS (stock 10X)	1X	50 mL
EDTA (stock 0.5 M)	2.5 mM	2.5 mL
FCS	2%	10 mL
NaN3 (stock 5%)	0.02%	2 mL
**Total**	N/A	**500 mL**

Store at 4°C for a maximum of 2 months.

**Table:** PrimeFlow RNA Fixation Buffer 1 (1 mL per sample)

Reagent	Final concentration	Amount
PrimeFlow RNA Fixation Buffer 1A	1X	500 μL
PrimeFlow RNA Fixation Buffer 1B	1X	500 μL
**Total**	N/A	**1 mL**

Prepare freshly directly before use. Keep at 4°C until use.

**Table:** PrimeFlow RNA Permeabilization Buffer (3 mL per sample)

Reagent	Final concentration	Amount
RNA Permeabilization Buffer (10X)	1X	300 μL
Water	N/A	2670 μL
RNase Inhibitors (100X)	1X	30 μL
**Total**	N/A	**3 mL**

Prepare freshly directly before use. Keep at 4°C until use.

**Table:** PrimeFlow RNA Fixation Buffer 2 (1 mL per sample)

Reagent	Final concentration	Amount
PrimeFlow RNA Fixation Buffer 2 (8X)	1X	125 μL
PrimeFlow RNA Wash Buffer	N/A	875 μL
**Total**	N/A	**1 mL**

Prepare freshly directly before use. Pre-warm to 20–25°C (RT) before use.

**Table:** PrimeFlow RNA Wash Buffer with RNase inhibitor (1 mL per sample)

Reagent	Final concentration	Amount
PrimeFlow RNA Wash Buffer	N/A	990 μL
RNase Inhibitors (100X)	1X	10 μL
**Total**	N/A	**1 mL**

Prepare freshly directly before use. Keep at 4°C until use.

**Table:** PrimeFlow Target Probe (100 μL per sample)

Reagent	Final concentration	Amount
PrimeFlow Target Probe	1X	5 μL
PrimeFlow Target Probe Diluent	N/A	95 μL
**Total**	N/A	**100 μL**

Prepare freshly directly before use. Target Probe Diluents needs to be pre-warmed to 40°C.

**Table:** PrimeFlow RNA Label Probe (100 μL per sample)

Reagent	Final concentration	Amount
PrimeFlow Target Probe (100X)	1X	1 μL
PrimeFlow RNA Label Probe Diluent	N/A	99 μL
**Total**	N/A	**100 μL**

Prepare freshly directly before use. RNA Label Probe Diluents needs to be pre-warmed to 40°C.

## Step-by-step method details

### Culture of bone marrow-derived macrophages

**Timing: 7 days**

This section describes the steps needed to differentiate macrophages from whole bone marrow of C57BL/6 WT mice.

1. Day 0: Plate 5 × 10^6^ bone marrow cells in 10 mL BMDM differentiation medium in a 10 cm dish.

***Note:*** A total number of 1–1.5 × 10^6^ BMDMs can be harvested per plate. This is important when planning the conditions of the co-culture seeding.

2. Day 5: Remove the BMDM differentiation medium and replace with 10 mL fresh BMDM differentiation medium.
3. Day 6: Remove the BMDM differentiation medium and replace with 10 mL fresh BMDM differentiation medium.
4. Day 7: BMDMs are ready to be used.
  a. For harvesting the cells, aspirate all supernatant.
  b. Wash the cells once with 1X PBS.
  c. Detach the cells from the culture dish using a cell scraper; transfer all cells into a 50 mL tube.
  d. Spin cells down at 300 × *g* for 10 min at 4°C.
  e. Resuspend cells in BMDM differentiation medium, count and adjust to 0.4 × 10^6^ cells/mL.

***Optional:*** Macrophages can be polarized into M1- or M2-like macrophages before co-culture seeding. Polarized macrophages can be used as controls, also without co-culture seeding. For M1-like polarization, treat the macrophages with LPS (20 ng/mL) and IFNγ (50 ng/mL); for M2-like polarization, treat macrophages with IL-4 (20 ng/mL). Polarization treatments should be applied for 16–24 h. Macrophages can either be treated on day 7 before harvest, or after seeding into 6-well plates.

**CRITICAL:** Please make sure that M-CSF is added to the BMDM differentiation medium freshly before use in the correct concentration. Lower concentrations can lead to reduced numbers of BMDMs on day 7 of the differentiation protocol.

### Culture of bone marrow-derived dendritic cells

**Timing: 7 days**

This section describes the steps needed to differentiate dendritic cells from whole bone marrow of C57BL/6 WT mice.

5. Day 0: Plate 6 × 10^6^ bone marrow cells in 10 mL BMDC differentiation medium in a 10 cm dish.
6. Day 3: Add 10 mL BMDC differentiation medium into each dish.
7. Day 5: Remove 10 mL culture medium.
  a. Spin down at 300 × *g* for 10 min at 4°C.
  b. Resuspend cells in 10 mL BMDC differentiation medium.
  c. Add the cells and medium back into the original culture plate (total volume: 20 mL).
8. Day 7: BMDCs are ready to be used.
  a. For harvesting the cells, collect cells from the supernatant; transfer into a 50 mL tube.
  b. Detach the cells from the culture dish using a cell scraper; add all cells into the 50 mL tube.
  c. Spin cells down at 300 × *g* for 10 min at 4°C.
  d. Resuspend cells in BMDC differentiation medium, count and adjust to 0.5 × 10^6^ cells/mL.

***Optional:*** Dendritic cells can be matured before co-culture seeding. Maturation of BMDCs can be achieved by treating with LPS (100 ng/mL). BMDCs should be treated for 16–24 h. Dendritic cells can either be treated on day 7 before harvest, or after seeding into 6-well plates.

**CRITICAL:** Please make sure that GM-CSF is added to the BMDC differentiation medium freshly before use in the correct concentration. Lower concentrations can lead to reduced numbers of BMDCs on day 7 of the differentiation protocol.

### ECP treatment and CellTrace Violet staining of splenocytes

**Timing: 2 h**

This step describes the treatment of donor splenocytes with Extracorporeal Photopheresis and the staining with CellTrace Violet. The analysis of phagocytosis of ECP-treated splenocytes by APCs upon co-culture is facilitated by fluorescence staining and the use of congenic mice. Specifically, wildtype C57BL/6 mice (CD45.2) are used for the culture of BMDMs and BMDCs. Congenic CD45.1 mice are used as donors for splenocytes, which undergo ECP treatment and CellTrace Violet staining. These are then co-cultured with BMDMs or BMDCs. The staining for CD45.1 and CD45.2 can discriminate between treated cells and phagocytes. If phagocytes become fluorescently labeled after co-culture, the phagocytes did engulf ECP-treated cells. Phagocytosis and tolerogenic polarization of APCs is analyzed after 48 h of co-culture.

9. Treatment of splenocytes with Extracorporeal Photopheresis.
  a. Euthanize donor mice (CD45.1) by cervical dislocation or a method approved by local animal ethic committees.
  b. Isolate the spleen.
  c. Mash the spleen through a 100 μm strainer into a 6 cm dish filled with 5 mL PBS.
  d. Spin down the cells at 300 × *g* for 5 min at 4°C.
  e. Resuspend cells in 2 mL ACK lysing buffer to lyse erythrocytes.
    i. Incubate at 20–25°C (RT) for 2 min.
    ii. Add 40 mL PBS and spin down the cells at 300 × *g* for 5 min at 4°C.
      ***Alternatives:*** Any other erythrocyte lysis buffer of your choice can used for this protocol.
  f. Resuspend splenocytes in cRPMI to 5 × 10^6^ cells/mL.
  g. Plate 12 mL cell suspension per dish into 10 cm plates.
  h. Add 8-methoxypsoralen (UVADEX) to a final concentration of 200 ng/mL.
    i. Incubate the cells for 30 min at 37°C in the dark.
  i. Apply UVA at a defined dose of 2.0 J/cm^2^ to the cells.
    i. The BS-02 UVA chamber (Opsytec) equipped with a UV-MAT can be used in dose-controlled mode.
  j. Wash the cells twice with 1X PBS.
  k. Discard the supernatant and resuspend the cells to 1 × 10^6^ cells/mL PBS in a 50 mL tube. PBS should be pre-warmed to 37°C.
    ***Optional:*** Other methods to induce apoptosis can be applied, depending on experimental setup. Details can be found in Braun LM et al.^1^
    ***Note:*** Some splenocytes should be kept untreated to control for cell death induction after 24 h. If calreticulin surface levels need to be analyzed, the splenocytes for this analysis do not necessarily need to undergo CTV staining. The staining with CTV is only important to track the phagocytic uptake of these cells after co-culture with phagocytes. The surface expression of calreticulin is analyzed on ECP-treated splenocytes, which were not co-cultured with phagocytes. CTV staining is only important to track the phagocytic uptake. However, the splenocytes can also be stained with CTV, as it does not interfere with calreticulin surface staining.
10. Label cells with CellTrace Violet.
  a. Dissolve one vial of CellTrace Violet (CTV) in 20 μL DMSO (5 mM stock).
  b. Dilute CTV stock 1:5 in 1X PBS.
  c. Stain splenocytes with 1 μM CTV. Vortex briefly.
  d. Incubate for 20 min at 37°C.
  e. Stop the staining by adding the double volume of cRPMI; spin cells down at 300 × *g* for 5 min at 20°C–25°C (RT).
  f. Wash cells twice with 30 mL cRPMI; spin cells down at 300 × *g* for 5 min at 20°C–25°C (RT).
  g. Resuspend cells in cRPMI and count; adjust cell concentration to 2 × 10^6^ cells/mL in cRPMI.

**CRITICAL:** In order to have enough CTV-stained ECP-treated splenocytes for co-culture, a loss of 30% of cells during staining should be calculated.

**CRITICAL:** The staining with CTV needs to be stopped with medium containing serum. The medium should contain at least 10% serum. Inefficient quenching results in increased loss of cells during the staining.

***Note:*** Machines for UVA application should be calibrated regularly if not operated in a dose-controlled mode to ensure application of the correct dosage of UVA to the splenocytes.

***Note:*** This protocol was adapted to analyze phagocytosis using CellTrace Violet. Any other intracellular staining can be used instead, e.g. CellTrace CFSE or Cell Proliferation Dye eFluor 670.

***Optional:*** If analysis of antigen presentation is planned, 5 × 10^6^ splenocytes can be pulse-treated with 10 μM Ovalbumin_257-264_ (chicken) for 2 h before co-culture seeding. Presentation of target cell antigens can be analyzed by staining MHC-bound SIINFEKL on APCs after co-culture.

### Co-culture seeding of APCs and ECP-treated CTV-stained splenocytes

**Timing: 1 h**

This step describes the co-culture seeding of APCs and ECP-treated CTV-stained splenocytes .The staining and the use of congenic mice facilitates analysis of phagocytosis of ECP-treated splenocytes by APCs upon co-culture. Phagocytosis and tolerogenic polarization of APCs is analyzed after 48 h of co-culture.

11. Seed 2 mL BMDMs or BMDCs per well into 6-well plates.
  a. BMDMs total cells count: 0.8 × 10^6^.
  b. BMDCs total cells count: 1 × 10^6^.

***Note:*** Three wells should be plated per condition to have one well each for FACS, PhosFlow and PrimeFlow.

12. Add 1 mL ECP-treated CTV-stained splenocytes per well.
  a. For “medium only control”, add 1 mL cRPMI per well.
  b. Splenocytes total cell count: 2 × 10^6^.
13. Spin the 6-well plates with the cells at 200 × *g* for 1 min at 20°C–25°C (RT).
14. Seed 2 × 10^6^ ECP-treated and untreated splenocytes in 2 mL cRPMI into 6-well plates for calreticulin staining. These cells do not need to be stained with CTV. Calreticulin surface levels were analyzed at 24 h after treatment with ECP.

***Optional:*** Calreticulin surface levels can additionally be analyzed at 48 h after treatment with ECP. In this case, seed more wells with ECP-treated and untreated cells.

15. Incubate the cells at 37°C, 5% CO_2_ for 48 h before analysis.

***Optional:*** In this step, inhibitors can be added into the co-culture to analyze molecular pathways and processes. Phagocytosis inhibitors were applied before addition of splenocytes; inhibitors for STAT6 and PPARγ were applied 1 h after addition of splenocytes to the APCs.

### Analysis of calreticulin surface expression by flow cytometry

**Timing: 2 h**

This step describes the staining of surface calreticulin levels on ECP-treated splenocytes 24 h after treatment with ECP. Calreticulin acts as an engulfment signal for phagocytic cells.^2^^,^^3^ Additionally, the viability of ECP-treated and untreated splenocytes is analyzed in this step. The analysis of splenocytes viability serves as a control for ECP-induced cell death. The investigators should see more than ∼65% dead cells at 24 h after ECP treatment. If splenocytes do not undergo cell death after treatment with ECP (compared to the untreated condition), this indicates that the treatment was not successful. The experiment should be repeated and the investigators should not use the co-culture for further analyses.

16. Transfer cells into a FACS tube, add 2 mL 1X PBS; spin cells down at 300 × *g* for 5 min at 4°C.
17. Discard the supernatant.
18. Wash cells once with 4 mL 1X PBS. Discard the supernatant.
19. Resuspend samples in 100 μL viability staining.
  a. Zombie NIR Fixable Viability Kit, 1:500 in PBS.
  b. Incubate 15 min at 4°C.
20. Wash cells twice with flow cytometry staining buffer (spin cells at 300 × *g* for 5 min at 4°C).
21. Resuspend samples in 50 μL diluted anti-CD16/32 antibody to block the Fc receptors:
  a. Anti-mouse CD16/32, 1:25 in flow cytometry staining buffer.
  b. Incubate 10 min at 4°C.
22. Directly add 50 μL surface staining antibody (total volume is 100 μL).
  a. Anti-Calreticulin, 1:100 in flow cytometry staining buffer (final dilution is 1:200).
  b. Incubate 20 min at 4°C.
23. Wash cells twice with flow cytometry staining buffer (spin cells at 300 × *g* for 5 min at 4°C).
24. Resuspend samples in 100 μL secondary antibody.
  a. Anti-rabbit PE, 1:50 in flow cytometry staining buffer.
  b. Incubate 20 min at 4°C.
25. Wash cells twice with flow cytometry staining buffer (spin cells at 300 × *g* for 5 min at 4°C).
26. Resuspend cells in flow cytometry staining buffer and keep the samples at 4°C until acquisition.

***Optional:*** In addition to viability analysis at the 24 h time point, the viability of splenocytes and calreticulin levels can be analyzed at 48 h after treatment with ECP.

***Note:*** The expected percentage of 65% dead cells at 24 h after treatment with ECP is based on experiments where splenocytes were used for ECP. If using other cell types or cell lines for ECP treatment or if other methods are used for cell death induction, the percentage of apoptotic cells might be different at 24 h post-treatment.

### FACS-based analysis of phagocytosis and BMDM polarization

**Timing: 3 h**

**Figure 1:**
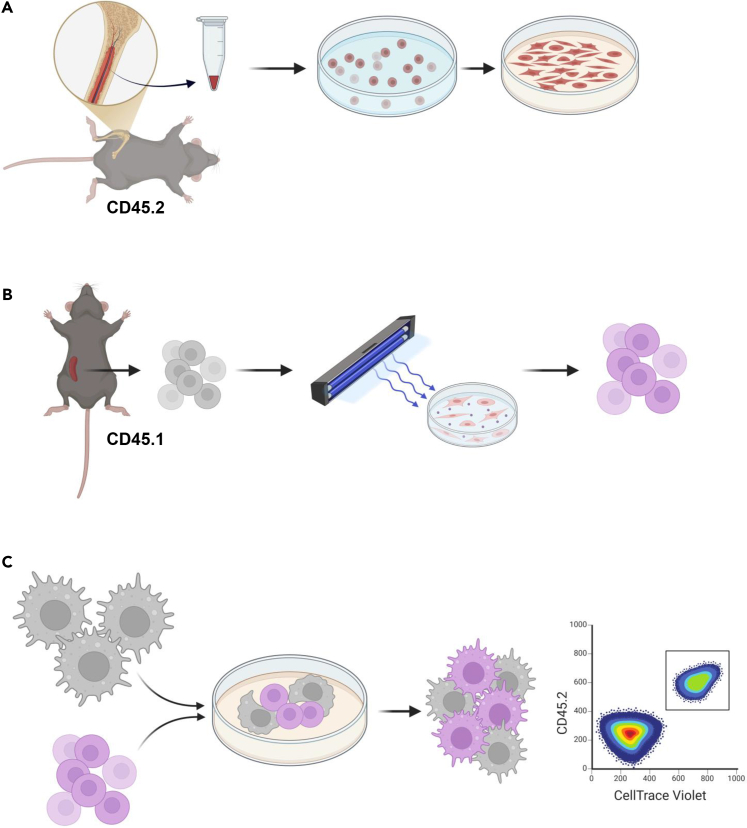
Graphical summary of the major steps of this protocol (A) Bone marrow-derived macrophages (BMDMs) and bone marrow-derived dendritic cells (BMDCs) are differentiated from murine bone marrow (CD45.2) for 7 days. (B) In the next step, splenocytes are isolated from congenic CD45.1 donor mice, and treated with 8-methoxypsoralen and UVA. Following, the cells are stained with the fluorescent dye CellTrace Violet. (C) Fluorescently labeled ECP-treated CD45.1 splenocytes are co-cultured with CD45.2 BMDMs or BMDCs for 48 h. Phagocytic cells are positive for CellTrace Violet after engulfment of ECP-treated splenocytes. The phagocytic uptake of ECP-treated splenocytes can be analyzed by flow cytometry using staining for congenic markers and CellTrace Violet signal. Further markers can be analyzed by flow cytometry.

This step describes the staining of surface antigens to discriminate between APCs and ECP-treated splenocytes by flow cytometry. The surface staining enables analysis of phagocytosis of ECP-treated cells by APCs through analysis of CellTrace Violet signal. Additionally, BMDMs are stained for surface markers and intracellular proteins to investigate macrophage polarization.^6^ A graphical summary of the protocol is shown in Figure 1.

27. BMDC analysis
  a. Harvest supernatant and scrape off adherent cells; transfer all cells into a 50 mL tube.
    ***Note:*** supernatant contains BMDCs and splenocytes, therefore both suspension and adherent cells need to be collected.
  b. Fill the tube with 1X PBS to 40 mL, spin cells down at 300 × *g* for 5 min at 4°C.
  c. Discard the supernatant.
  d. Distribute cells into a 96-well round bottom plate for staining.
  e. Spin down cells at 500 × *g* for 3 min at 4°C.
  f. Discard supernatant.
  g. Resuspend samples in 100 μL viability staining.
    i. Zombie NIR Fixable Viability Kit, 1:500 in PBS.
    ii. Incubate 15 min at 4°C.
  h. Wash cells twice with flow cytometry staining buffer (spin cells at 500 × *g* for 3 min at 4°C).
  i. Resuspend samples in 50 μL diluted anti-CD16/32 antibody to block the Fc receptors:
    i. Anti-mouse CD16/32, 1:25 in flow cytometry staining buffer.
    ii. Incubate 10 min at 4°C.
  j. Directly add 50 μL surface staining antibody mix (total volume is 100 μL).
    i. Anti-mouse CD45.1 (FITC), anti-mouse CD45.2 (BV711); both 1:50 in flow cytometry staining buffer (final dilution is 1:100).
    ii. Incubate 30 min at 4°C.
  k. Wash cells twice with flow cytometry staining buffer (spin cells at 500 × *g* for 3 min at 4°C).
  l. Resuspend cells in 200 μL flow cytometry staining buffer and keep at 4°C in the dark until acquisition.
    ***Optional:*** The surface markers CD80 and CD86 can be stained together with CD45.1 and CD45.2 to analyze BMDCs maturation.
28. BMDM analysis.
  a. Discard the supernatant and wash the BMDM layer once with PBS.
    ***Optional:*** Cells in the supernatant can be collected if interested. BMDMs adhere to the plate and need to be scraped off for analysis.
  b. Scrape off BMDMs using a cell scraper.
  c. Transfer cells into a 50 mL tube into 40 mL 1X PBS; spin cells down at 300 × *g* for 5 min at 4°C.
  d. Discard the supernatant.
  e. Distribute cells into a 96-well round bottom plate for staining.
  f. Spin down cells at 500 × *g* for 3 min at 4°C.
  g. Discard supernatant.
  h. Resuspend samples in 100 μL viability staining.
    i. Zombie NIR Fixable Viability Kit, 1:500 in PBS.
    ii. Incubate 15 min at 4°C.
  i. Wash cells twice with flow cytometry staining buffer (spin cells at 500 × *g* for 3 min at 4°C).
  j. Resuspend samples in 50 μL diluted anti-CD16/32 antibody to block the Fc receptors:
    i. Anti-mouse CD16/32, 1:25 in flow cytometry staining buffer.
    ii. Incubate 10 min at 4°C.
  k. Directly add 50 μL surface staining antibody mix (total volume is 100 μL).
    i. Anti-mouse CD45.1 (FITC), anti-mouse CD45.2 (BV711), anti-mouse CD206 (PE), anti-mouse CD301 (PE-Cy7); all 1:50 in flow cytometry staining buffer (final dilution is 1:100).
    ii. Incubate 30 min at 4°C.
  l. Wash cells twice with flow cytometry staining buffer (spin cells at 500 × *g* for 3 min at 4°C).
  m. Resuspend cells in 200 μL fixation/permeabilization solution (BD Cytofix/Cytoperm).
    i. Incubate 40 min at 4°C.
    ii. Spin cells down at 750 × *g* for 3 min at 4°C.
  n. Wash cells twice with 200 μL 1X BD Perm/Wash Buffer; spin at 750 × *g* for 3 min at 4°C.
  o. Resuspend cells in 100 μL intracellular stain.
    i. Anti-mouse/human Arginase 1 (APC); 1:100 in 1X BD Perm/Wash.
    ii. Incubate 30 min at 4°C.
    iii. Wash cells twice with 200 μL 1X BD Perm/Wash Buffer.
  p. Resuspend cells in 150 μL 1X BD Perm/Wash Buffer and keep at 4°C in the dark until acquisition.
    ***Note:*** The viability stain and the fluorophores reported in this protocol are just suggestions and can be changed according to the availability of antibodies or machines used for analysis.
    ***Alternatives:*** In this protocol, a monoclonal antibody targeting mouse CD16/32 was used to block the Fc receptors. Other commercial products can be used instead (e.g. TruStain FcX (anti-mouse CD16/32, BioLegend), FcR Blocking Reagent (Miltenyi Biotec) or Purified Rat Anti-Mouse CD16/CD32 (Mouse BD Fc Block, BD Pharmingen)).
    **Pause point:** The BMDMs are fixed and can therefore be kept at 4°C for about 24 h before acquisition.

### FACS-based analysis of STAT6 phosphorylation in BMDMs after co-culture with ECP-treated splenocytes

**Timing: 2 h**

This step describes the staining of surface antigens to identify BMDMs by flow cytometry. The surface staining enables analysis of phagocytosis of ECP-treated cells by APCs through analysis of CellTrace Violet signal. Additionally, phosphorylated STAT6 is analyzed in this step.

29. Discard the supernatant and wash the BMDM layer once with PBS.
30. Scrape off BMDMs using a cell scraper.
31. Transfer cells into a 50 mL tube into 40 mL 1X PBS; spin cells down at 300 × *g* for 5 min at 4°C.
32. Discard the supernatant.
33. Distribute cells into a 96-well round bottom plate for staining.
  a. Spin down cells at 500 × *g* for 3 min at 4°C.
  b. Discard supernatant.
34. Resuspend samples in 100 μL viability staining.
  a. Zombie NIR Fixable Viability Kit, 1:500 in PBS.
  b. Incubate 10 min at 4°C.
35. Wash cells twice with flow cytometry staining buffer (spin cells at 500 × *g* for 3 min at 4°C).
36. Resuspend samples in 50 μL diluted anti-CD16/32 antibody to block the Fc receptor:
  a. Anti-mouse CD16/32, 1:25 in flow cytometry staining buffer.
  b. Incubate 5 min at 4°C.
37. Directly add 50 μL surface staining antibody mix (total volume is 100 μL).
  a. Anti-mouse CD45.2 (PE); 1:50 in flow cytometry staining buffer (final dilution is 1:100).
  b. Incubate 15 min at 4°C.
38. Wash cells twice with flow cytometry staining buffer (spin cells at 500 × *g* for 3 min at 4°C).
39. Resuspend cells in 200 μL pre-warmed 1X Lyse/Fix Buffer.
  a. Incubate 10 min at 37°C.
40. Spin cells at 500 × *g* for 3 min at 4°C; discard the supernatant.
41. Wash cells once with flow cytometry staining buffer.
42. Resuspend cells in 200 μL ice-cold Perm Buffer III.
  a. Incubate 30 min at 4°C.
43. Spin cells at 500 × *g* for 3 min at 4°C; discard the supernatant.
44. Wash cells twice with BD Stain Buffer.
45. Resuspend samples in 100 μL antibody mix.
  a. Anti-mouse STAT6 (pY641) AlexaFluor 488, 1:5 in BD Stain Buffer.
  b. Incubate 30 min at 4°C.
46. Wash cells twice with BD Stain Buffer.
47. Resuspend cells in 150 μL BD Stain Buffer and keep at 4°C in the dark until acquisition. Samples should be acquired directly after staining.

***Note:*** The viability stain and the fluorophores reported in this protocol are just suggestions and can be changed according to the availability of antibodies or machines used for analysis.

### FACS-based analysis of *Adipoq* gene expression in BMDMs after co-culture with ECP-treated splenocytes

**Timing: 2 days**

This step describes the staining of surface antigens to identify BMDMs by flow cytometry. The surface staining enables analysis of phagocytosis of ECP-treated cells by APCs through analysis of CellTrace Violet signal. The hybridization of a gene-specific target probe to identify changes of gene expression in single cells is explained in this step.

48. Discard the supernatant and wash the BMDM layer once with PBS.
49. Scrape off BMDMs using a cell scraper.
50. Transfer cells into a 50 mL tube into 40 mL 1X PBS; spin cells down at 300 × *g* for 5 min at 4°C.
51. Discard the supernatant.
52. Distribute cells into a 96-well round bottom plate for staining.
  a. Spin down cells at 500 × *g* for 3 min at 4°C.
  b. Discard supernatant.
53. Resuspend samples in 100 μL viability staining.
  a. Zombie NIR Fixable Viability Kit, 1:500 in PBS.
  b. Incubate 10 min at 4°C.
54. Wash cells twice with flow cytometry staining buffer (spin cells at 500 × *g* for 3 min at 4°C).
55. Resuspend samples in 50 μL diluted anti-CD16/32 antibody to block the Fc receptors:
  a. Anti-mouse CD16/32, 1:25 in flow cytometry staining buffer.
  b. Incubate 5 min at 4°C.
56. Directly add 50 μL surface staining antibody mix (total volume is 100 μL).
  a. Anti-mouse CD45.2 (PE); 1:50 in flow cytometry staining buffer (final dilution is 1:100).
  b. Incubate 15 min at 4°C.
57. Transfer cells into 1.5 mL tubes; only use the tubes provided by the PrimeFlow Kit.
58. Add 1 mL flow cytometry staining buffer; spin cells 800 × *g* for 5 min at 4°C.
  a. Discard the supernatant.
  b. Repeat the washing step and discard the supernatant.
59. Add 1 mL PrimeFlow RNA Fixation Buffer 1 into each sample; invert the tube to mix.
  a. Incubate 30 min at 4°C.
  b. Spin down cells at 800 × *g* for 5 min at 4°C.
  c. Resuspend cells in the residual volume.
60. Add 1 mL PrimeFlow RNA Permeabilization Buffer with RNase inhibitor into each sample.
  a. Spin down cells at 800 × *g* for 5 min at 4°C.
  b. Repeat this step twice for a total of 3 washes with RNA permeabilization Buffer.
61. Add 1 mL PrimeFlow RNA Fixation Buffer 2 into each sample; invert the tube to mix.
  a. Incubate 60 min at 20°C–25°C (RT) in the dark.
    **CRITICAL:** Do not incubate on ice.
  b. Spin down cells at 800 × *g* for 5 min at 4°C.
  c. Aspirate all but 100 μL of the supernatant; vortex the samples to resuspend the cells.
62. Add 1 mL PrimeFlow RNA Wash Buffer to each sample; invert the tube to mix.
  a. Spin down cells at 800 × *g* for 5 min at 4°C.
  b. Aspirate all but 100 μL of the supernatant; vortex the samples to resuspend the cells.
  c. Repeat this step for a total of 2 washes; cells should remain in 100 μL.
63. Pre-warm the Target Probe Diluent to 40°C.
  a. Dilute the target probe(s) 1:20 in pre-warmed Target Probe Diluent.
64. Add 100 μL diluted target probe directly into the cell suspension; vortex.
  a. Incubate 2 h at 40°C; vortex for 3 seconds after 1 h.
65. Add 1 mL PrimeFlow RNA Wash Buffer to each sample; invert the tube to mix.
  a. Spin down cells at 800 × *g* for 5 min at 4°C.
  b. Aspirate all but 100 μL of the supernatant; vortex the samples to resuspend the cells.
66. Add 1 mL PrimeFlow RNA Wash Buffer with RNase inhibitor to each sample; invert the tube to mix.
  a. Spin down cells at 800 × *g* for 5 min at 4°C.
  b. Aspirate all but 100 μL of the supernatant; vortex the samples to resuspend the cells.
67. Store the samples at 4°C until the next morning. Storage should not exceed 16 h.

**Pause point:** The samples can be kept at 4°C until the next morning (step 67) to split the protocol into two days, as it might be too long for one day. However, step 67 can also be skipped and the protocol can be directly continued with step 68. The protocol has to be continued until step 77 in this case, as there is no other possible pause point.

68. Pre-warm samples and PrimeFlow RNA Wash Buffer to 20°C–25°C (RT).
69. Pre-warm PrimeFlow PreAmp Mix, PrimeFlow RNA Amp Mix and PrimeFlow RNA Label Probe Diluent to 40°C.
70. Add 100 μL of PrimeFlow PreAmp Mix directly into the cell suspension; vortex for 3 seconds.
  a. Incubate 90 min at 40°C.
71. Add 1 mL PrimeFlow RNA Wash Buffer to each sample; invert the tube to mix.
  a. Spin down cells at 800 × *g* for 5 min at 20°C–25°C (RT).
  b. Aspirate all but 100 μL of the supernatant; vortex the samples to resuspend the cells.
  c. Repeat this step for a total of 3 washes; cells should remain in 100 μL.
72. Add 100 μL PrimeFlow RNA Amp Mix directly into the cell suspension; vortex for 3 seconds.
  a. Incubate 90 min at 40°C.
73. Add 1 mL PrimeFlow RNA Wash Buffer to each sample; invert the tube to mix.
  a. Spin down cells at 800 × *g* for 5 min at 20°C–25°C (RT).
  b. Aspirate all but 100 μL of the supernatant; vortex the samples to resuspend the cells.
  c. Repeat this step for a total of 2 washes; cells should remain in 100 μL.
74. Dilute RNA label probes 1:100 in PrimeFlow Label Probe Diluent.
  a. Add 100 μL diluted Label Probes directly into the cell suspension for each sample; vortex for 3 seconds.
  b. Incubate 60 min at 40°C.
75. Add 1 mL PrimeFlow RNA Wash Buffer to each sample; invert the tube to mix.
  a. Spin down cells at 800 × *g* for 5 min at 20°C–25°C (RT).
  b. Aspirate all but 100 μL of the supernatant; vortex the samples to resuspend the cells.
  c. Repeat this step for a total of 2 washes; cells should remain in 100 μL.
76. Add 1 mL PrimeFlow RNA Storage Buffer to each sample; invert to mix.
  a. Spin down cells at 800 × *g* for 5 min at 20°C–25°C (RT).
  b. Aspirate all but 100 μL of the supernatant; vortex the samples to resuspend the cells.
77. Transfer cells into polystyrene tubes, add 100 μL PrimeFlow RNA Storage Buffer into each sample and keep samples at 4°C until acquisition.

***Note:*** The viability stain and the fluorophores reported in this protocol are just suggestions and can be changed according to the availability of antibodies or machines used for analysis. Target probes for other genes can be added into the panel; target gene analysis is mainly limited by the availability of fluorophores by the manufacturer.

***Alternatives:*** A Type 1 target probe was used in these experiments, as the expression levels of *Adipoq* were unknown. In addition, type 4, type 6 and type 10 target probes are available for the detection of transcripts. The target probes are all labeled with different fluorochromes and should be chosen based on target gene expression and fluorochromes used to detect other markers.

***Note:*** In addition to surface proteins, intracellular and intranuclear proteins can be stained with the PrimeFlow staining protocol. See manufacturer’s instructions for the detailed procedure.

### Data analysis

**Timing: 1–2 h**

**Figure 2:**
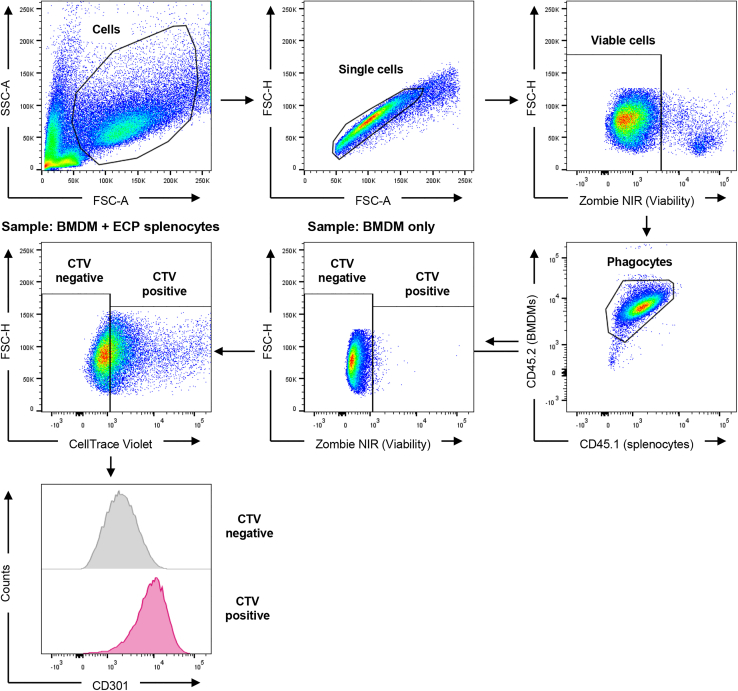
Exemplary gating strategy to analyze phagocytosis of ECP-treated splenocytes and BMDM polarization This gating strategy shows the analysis of BMDMs. The first gate includes all cells (mainly viable BMDMs); small cells and debris can be excluded. BMDMs should form a uniform population. Next, the gate is set on single cells, followed by gating on viable cells. To further purify the population, the gate is set on CD45.2^+^ and CD45.1^–^ cells. CD45.2 is expressed by BMDMs, whereas CD45.1 is expressed by ECP-treated splenocytes. Thereby, the analysis is purely focusing on BMDMs. In the next step, gates are defined for CTV-positive and CTV-negative cells. These gates should be defined based on CTV expression in samples, which were not co-cultured with ECP-treated splenocytes (BMDMs only). These cells are all negative for CTV. Following, fluorescence intensity for CD301, CD206, Arginase 1, STAT6 or *Adipoq* can be analyzed in CTV-positive vs. CTV-negative cells.

This part of the protocol describes the analysis of the primary data collected by flow cytometry. An exemplary gating strategy is shown in Figure 2.

78. Analysis of flow cytometry data.
  a. Export acquired flow data as .fcs files and open in the FlowJo analysis software.
  b. Gate on all cells, then on single cells.
  c. Gate on viable cells and exclude all dead cells.
  d. Analysis of phagocytosis.
    i. Gate on CD45.2^+^ antigen-presenting cells. A gate for CD45.1^+^ cells can be added; these can be excluded from the analysis.
    ii. Gate on CellTrace Violet positive cells. These cells represent APCs with engulfed ECP-treated splenocytes.
      ***Note:*** This analysis focuses on CD45.2^+^ antigen-presenting cells, as their phenotype should be analyzed after co-culture with CD45.1^+^ ECP-treated splenocytes. CD45.2^+^ APCs engulf ECP-treated splenocytes.
  e. Analysis of STAT6 phosphorylation.
    i. Gate on CD45.2^+^ antigen-presenting cells.
    ii. Gate on CellTrace Violet positive cells. These cells represent APCs with engulfed ECP-treated splenocytes.
    iii. Calculate MFI for pSTAT6 from CTV^+^ vs. CTV^–^ cells or CD45.2^+^ cells with ECP-treated splenocytes vs. without (medium only).
  f. Analysis of *Adipoq* gene expression.
    i. Gate on CD45.2^+^ antigen-presenting cells.
    ii. Gate on CellTrace Violet positive cells. These cells represent APCs with engulfed ECP-treated splenocytes.
    iii. Calculate MFI for *Adipoq* from CTV^+^ vs. CTV^–^ cells or CD45.2^+^ cells with ECP-treated splenocytes vs. without (medium only).
  g. Analysis of calreticulin surface level.
    i. Calculate MFI for calreticulin or calreticulin positive cells from all viable cells after ECP treatment.
79. Data visualization and statistical analysis.
  a. Paste the results from the flow data into GraphPad Prism, test for normality using the Shapiro-Wilk test and perform suitable tests to analyze statistical differences.

**Pause point:** Researchers can pause the protocol after data acquisition and analysis can be done later.

***Note:*** We have used the FlowJo software to analyze the flow cytometry data. If not available, any other analysis software for flow cytometry data can be used instead.

***Alternatives:*** We have chosen FlowJo and Prism to analyze flow cytometry data and to perform data visualization and statistical testing. Any other software suitable for flow cytometry data analysis can be used. Statistical analysis and data visualization can be done with other software, depending on available resources.

## Expected outcomes

The successful analysis of phagocytosis should result in CellTrace Violet-stained CD45.2^+^ APCs. Viability staining and exclusion of CD45.1^+^ cells can further remove apoptotic splenocytes. Phagocytes with engulfed ECP-treated splenocytes become positive for CellTrace Violet.^1^ In addition, CD206, CD301 and Arginase 1 can indicate if BMDMs are polarized towards an immunosuppressive phenotype after co-culture with ECP-treated splenocytes. Based on our results we know that co-culturing ECP-treated splenocytes with BMDMs causes upregulation of Arginase 1 and phosphorylation of STAT6 in BMDMs. Moreover, the M2-like BMDMs upregulate the expression of *Adipoq* upon co-culture with ECP-treated splenocytes. Moreover, we found that the splenocytes enhance the expression of calreticulin on their surface, which triggers the phagocytic clearance of these cells. All expected outcomes can be found in Braun LM et al.^1^

## Limitations

This protocol was developed to specifically understand if ECP-treated splenocytes are phagocytosed by bone marrow-derived macrophages or bone marrow-derived dendritic cells. We had used splenocytes based on our preclinical *in vivo* models, in which we used ECP-treated splenocytes to resolve inflammation following treatment with immune checkpoint inhibitors. The main focus was to understand if these apoptotic cells are cleared by phagocytes and if the phenotype of these phagocytes is changed after the phagocytic uptake. This is a setting, which was specific to our project, but also other APCs could be used as phagocytic cells, or the protocol could be adapted to monitor the clearance of tumor cells by macrophages.^7^^,^^8^

In addition, we only report the use of ECP for cell death induction in this protocol, but cell death could also be induced by pre-treating the splenocytes with chemotherapy.^1^

The major limitation of this protocol is the need to use the PrimeFlow Kit for the analysis of target gene expression in single cells. We and others have verified that target gene expression can be efficiently analyzed after probe hybridization.^9^^,^^10^ However, the number of possible genes per reaction is limited by available target probe types. In addition, the efficacy of target probe hybridization is dependent on stable temperatures during the hybridization process and unstable temperatures can significantly reduce probe hybridization and signal amplification. Further, the number of target antigens for analysis is limited by the PrimeFlow Assay, as not all fluorochromes are compatible with the assay. The manufacturer’s instructions contains a list all fluorochromes which are reported to work with the assay or which are not recommended.

## Troubleshooting

### Problem 1

The purity of BMDMs or BMDCs is low after 7 days of culture/differentiation.

### Potential solution

The protocols described here should yield >90% purity of BMDMs and BMDCs after the 7 d differentiation. It is critical that the cytokines for BMDM and BMDC generation are added freshly into the culture medium and that the cytokines are prepared according to manufacturer’s instructions. The LOT-specific instructions for cytokine reconstitutions can be downloaded after the purchase of the product. When harvesting these cells, only scrape them off the plate and do not add trypsin to the cells.

### Problem 2

ECP-treated splenocytes are not stained with CellTrace Violet.

### Potential solution

Proteins quench the staining with CellTrace Violet. It is therefore crucial that the cells are washed with PBS before staining and that the staining is done in PBS w/o any addition of protein. The steps need to be followed as outlined in this protocol. To make sure that CellTrace Violet staining was successful, an aliquot of the cells can be analyzed before setting up the co-culture with APCs.

### Problem 3

More than 30% of splenocytes are lost during CellTrace Violet staining.

### Potential solution

During CellTrace Violet staining, it is commonly seen to lose up to 30% of the splenocytes. If more cells are lost during the staining process, the steps need to be optimized. Higher concentrations of CellTrace Violet or incubation times longer than what is given in the protocol can cause overstaining, cell death and loss of cells. Additionally, incubation times, temperatures and centrifugation steps should not be modified to ensure successful CellTrace Violet staining with minimal loss of cells.

### Problem 4

No staining is observed for CD45.1, CD45.2 and Arginase 1.

### Potential solution

All targets used for staining in this protocol are generally well established and easy to stain with commercially available antibodies. If surface markers are found negative, it should be verified that the antibodies were added into the staining mix. CD45.1 is only found on a small percentage of the cells, as it marks ECP-treated splenocytes, which undergo apoptosis within 24 h after treatment.

The staining for Arginase 1 is dependent on efficient permeabilization of the cells. Make sure to stick to the times for fixation and permeabilization to ensure efficient permeabilization of the cells for intracellular staining. The products and timing described in this protocol are sufficient for Arginase 1 staining. Substitutions with other products and changes of the protocol should be critically evaluated. IL-4 treated BMDMs (M2-like) can be used to assess the Arginase 1 staining efficacy.

### Problem 5

No staining is observed for pSTAT6.

### Potential solution

The staining for pSTAT6 is established as described in this protocol. Reagents for fixation or permeabilization might be substituted, but the staining efficacy needs to be critically evaluated. Make sure that you stick to the temperatures and incubation times for pSTAT6 staining as described in this protocol.

### Problem 6

No staining is observed for *Adipoq*.

### Potential solution

The analysis of *Adipoq* expression in single cells using a target-specific probe is established according to the protocol described here. It is crucial to follow precisely all steps as described here and to use all reagents provided by the manufacturer. Please add RNase inhibitor into the buffers as described in the protocol to prevent RNA degradation. In addition, unstable temperatures during probe hybridization and amplification can yield in poor target probe hybridization and low signals. The label probe type needs to be chosen based on the target gene expression. According to the manufacturer, type 1 probes (Alexa Fluor 647) and type 10 probes (Alexa Fluor 568) have a high sensitivity and are preferred for low or unknown expression levels. Type 4 probes (Alexa Fluor 488) and type 6 probes (Alexa Fluor 750) have an intermediate to low sensitivity and are best for the analysis of medium to high expression levels.

### Problem 7

Cells do not undergo cell death after treatment with ECP.

### Potential solution

Splenocytes treated with 8-MOP and UVA, which is ECP, undergo detectable cell death within 24 h after treatment. If cells do not undergo apoptosis, verify that UVA is applied correctly to the cells. The UVA chamber should be operated in a dose-controlled mode. The UVA sensor needs to be calibrated regularly. 8-MOP should not be kept in the dark to prevent loss of efficiency.

## Resource availability

### Lead contact

Further information and requests for resources and reagents should be directed to and will be fulfilled by the lead contact, Robert Zeiser (robert.zeiser@uniklinik-freiburg.de).

### Technical contact

Questions regarding the technical specifics of performing the protocol should be directed to the technical contact, Lukas Braun (lukas.braun@uniklinik-freiburg.de).

### Materials availability

This study did not generate new unique reagents.

### Data and code availability

This study did not generate any unique datasets or codes.

## Acknowledgments

Parts of the figures and the graphical abstract were created using Biorender.com. This study was supported by grants from the Deutsche Forschungsgemeinschaft (DFG, German Research Foundation): project-ID 441891347—SFB1479, project-ID 259373024—TRR167, and project-ID 256073931—CRC1160 (all to R.Z.). This study was further supported by the European Union: EU proposal number ERC-2022-ADG, project 101094168—AlloCure (ERC Advanced grant to R.Z.); EU project: project-ID 101119855—exTra; ERANET Transcan—PIXEL; and ERA-NET Transcan—SmartCART (all to R.Z.). Further, the study received support from CIBSS—EXC-2189—project-ID 390939984, the Deutsche Krebshilfe (70114655), the Jose-Carreras Leukemia foundation (DJCLS 09R/2022), the Leukemia & Lymphoma Society (7030-23), and the German Cancer Consortium (DKTK), all to R.Z.

We thank the Lighthouse Core Facility at the University Medical Center Freiburg. The Lighthouse Core Facility is funded in part by the Medical Faculty, University of Freiburg (project numbers 2023/A2-Fol; 2021/B3-Fol), the DKTK, the Mertelsmann Foundation, and the DFG (project number 450392965). We thank the staff of the animal facility at the University Medical Center Freiburg for their support.

## Author contributions

Conceptualization, L.M.B. and R.Z.; methodology, L.M.B. and R.Z.; investigation, L.M.B.; formal analysis, L.M.B.; writing – original draft, L.M.B. and R.Z.; writing – review and editing, L.M.B. and R.Z.; funding acquisition, R.Z.; supervision, R.Z.

## Declaration of interests

R.Z. has received honoraria from Novartis, Incyte, Sanofi, Neovii, and Mallinckrodt. All honoraria and support were outside this work.
